# Use of Vein Conduit and Isolated Nerve Graft in Peripheral Nerve Repair: A Comparative Study

**DOI:** 10.1155/2014/587968

**Published:** 2014-10-27

**Authors:** Imran Ahmad, Md. Sohaib Akhtar

**Affiliations:** Post Graduate Department of Burns, Plastic and Reconstructive Surgery, JNMC, AMU, Aligarh, Uttar Pradesh 202002, India

## Abstract

*Aims and Objectives.* The aim of this study was to evaluate the effectiveness of vein conduit in nerve repair compared with isolated nerve graft. *Materials and Methods.* This retrospective study was conducted at author's centre and included a total of 40 patients. All the patients had nerve defect of more than 3 cm and underwent nerve repair using nerve graft from sural nerve. In 20 cases, vein conduit (study group) was used whereas no conduit was used in other 20 cases. Patients were followed up for 2 years at the intervals of 3 months. *Results.* Patients had varying degree of recovery. Sensations reached to all the digits at 1 year in study groups compared to 18 months in control group. At the end of second year, 84% patients of the study group achieved 2-point discrimination of <10 mm compared to 60% only in control group. In terms of motor recovery, 82% patients achieved satisfactory hand function in study group compared to 56% in control group (*P* < .05). *Conclusions.* It was concluded that the use of vein conduit in peripheral nerve repair is more effective method than isolated nerve graft providing good sensory and motor recovery.

## 1. Introduction

Successful repair of peripheral nerve injury remains a difficult task for the reconstructive surgeons. A small nerve gap can be repaired primarily which is one of the best methods of repair [1]. A large nerve gap may require grafts. The various available options for grafting are autologous nonvascularised nerve graft, autologous vascularised nerve grafts, interposition of venous or arterial segments, or use of muscle or synthetic conduits [2]. Cable grafts for large nerve defects have been universally used for nerve repair [3, 4].

There are various available conduits that have been used for peripheral nerve repair. Use of vein as conduit is well-described in the literature [5]. Many investigators have used vein grafts for peripheral nerve repair that can be used alone or packed with muscle fibres [6, 7]. Vein conduit helps in nerve regeneration by preventing sprouting of nerve fibres at the neurorraphy sites [8, 9]. Besides, there are neurotrophic factors released from the endothelial layer of the vein that provides a more favourable environment for regeneration [10]. Vein conduit also leads to lower inflammatory cells to migrate, higher rate of axonal regeneration under neurotropism, and a thinner epineurium to regenerate [11–13].

In this series, we have used autologous sural nerve for the repair of peripheral nerve with and without vein conduit. To the best of our knowledge, currently there is no study that focuses on comparison between use of nerve graft with conduit and isolated nerve graft.

## 2. Materials and Methods

This retrospective study included a total of 40 patients who underwent nerve repair between November 2010 and January 2013. The study was approved by the Ethics Committee of the Hospital and informed consent was taken from each patient. All the patients had nerve defect of more than 3 cm and underwent nerve repair using autologous nerve graft from sural nerve. In 20 cases, we used autologous short saphenous vein as conduit (study group) while in other 20 cases, no conduit was used. Information regarding age, etiology of the defects, duration of injury, types of nerve and repair, nerve defect, distance between proximal nerve end and tip of middle finger, associated vascular injury, and comorbidity were recorded from patients' medical records. Participating patients were matched with a randomly selected cohort of control patients with nerve defects, according to age, etiology of the defects, duration of injury, types of nerve repair, nerve defect, distance between proximal nerve end and tip of middle finger, associated vascular injury, tendon injury, and comorbidity, who were treated by nerve graft without vein conduit (Table 1). Associated tendon or vessel injury was repaired in the standard way. All patients were operated under general anesthesia in supine position with tourniquet control.

### 2.1. Surgical Technique

**Figure 1:**
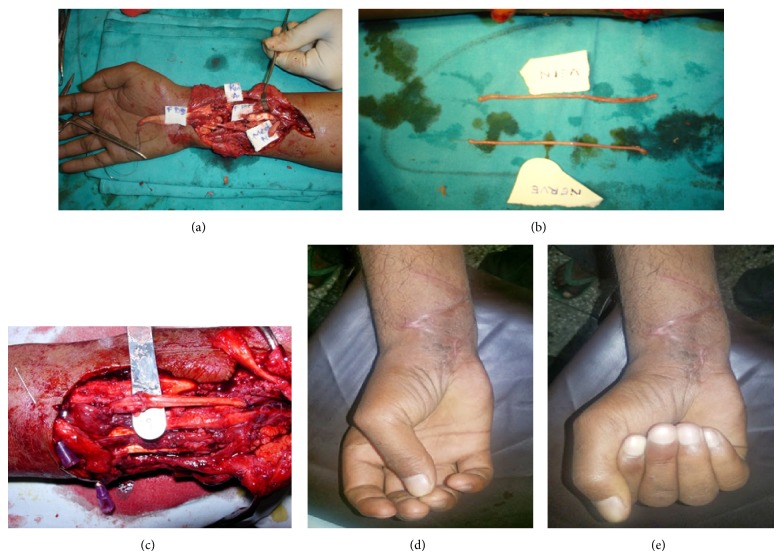
(a) Photograph showing median nerve gap. Associated tendon injury is visible. (b) Sural nerve and short saphaneous vein harvested. (c) Graft inerted into the defect after the nerve was packed within the vein. (d) and (e) Follow-up photograph showing good functional recovery.

**Figure 2:**
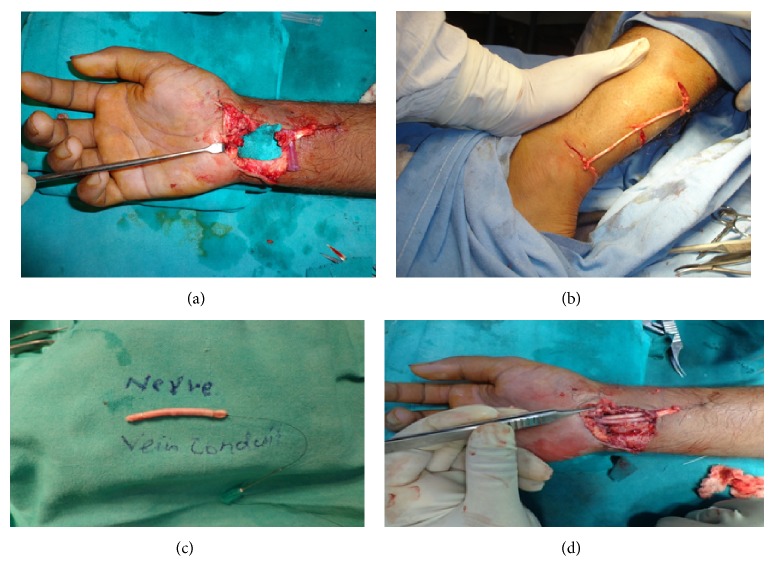
(a) Photograph showing median nerve defect. (b) Harvesting of sural nerve and short saphaneous vein through the same incision. (c) Sural nerve packed within the short saphaneous vein after turning on itself. (d) Graft inserted into the defect and anastomosis done.

**Figure 3:**
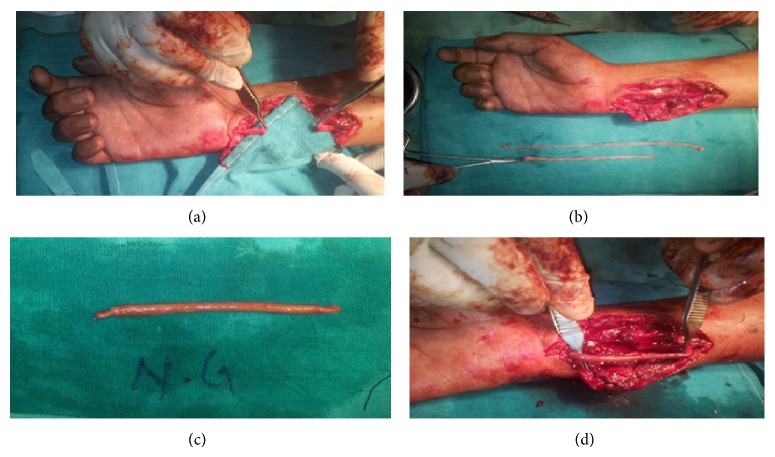
(a) Photograph showing ulnar nerve defect right hand. (b) Sural nerve and short saphaneous vein harvested. (c) Sural nerve packed within the short saphaneous vein after turning on itself. (d) Graft put into the defect and anastomosis done.

Following steps were performed (Figures 1, 2, and 3).

1. There is exploration of the affected nerve under magnification.
2. Both the ends were identified.
3. Neuroma or scar, if present, was excised up to the healthy fascicles while in case of fresh injury, ends were trimmed till bleeding and then defect was measured.
4. The fascicles from each end were properly oriented and aligned.
5. Sural nerve graft and short saphanous vein were harvested through same incisions.
6. Vein was turned inside out.
7. Sural nerve was turned on itself and packed within the vein (2–5 times depending on diameter of the nerve).
8. Both the ends were excised to get freshly cut ends.
9. Then graft was put into the defect.
10. Tension free repair was done taking interrupted sutures between epineurium and vein wall.
11. Skin closure was done.
12. Limb was immobilized in slightly flexed position.

Patients were followed up for 2 years at intervals of 3 months. At the end of 2 years, following scales were used for final evaluation of sensory and motor recovery.

1. Scale for 2-point discrimination:
   normal = 0–5 mm;
   fair = 6–10 mm;
   poor = 11–15 mm;
   protective sensation = 1 point;
   anesthetic = no point.
2. Semmes-Weinstein monofilament test:
   normal = 2.83;
   diminished = 3.61;
   diminished protective = 4.31;
   loss of protective = 4.56.
3. Pain/discomfort evaluation:
   0 = function hindered;
   1 = disturbed;
   2 = moderate;
   3 = none (normal).
4. Power grading of the affected muscles:
   grade 0: complete paralysis;
   grade 1: flicker of contraction present;
   grade 2: active movement with gravity eliminated;
   grade 3: active movement against gravity;
   grade 4: active movement against gravity and some resistance;
   grade 5: normal power.
5. Medical research scale (MRC scale):
   0 = no atrophy;
   1 = mild atrophy;
   2 = moderate atrophy;
   3 = severe atrophy.

While evaluating sensation at the finger tips, the other uninjured nerve was blocked by local anesthesia.

MRC grading was done by assessing muscle strength and size of the first dorsal interossei (for ulnar nerve evaluation) and flexor pollicis brevis for median nerve evaluation. Atrophy of these muscles was graded as 0, 1, 2, and 3.

All the patients underwent nerve conduction study on follow-up.

## 3. Results

All the patients were evaluated in terms of sensory and motor recovery. Two-point discrimination (2-PD), Semmes-Weinstein monofilament test (SW test) and power of involved muscles, and MRC scale were recorded. Pain/discomfort evaluation was performed for cold intolerance.

Sensations reached to all the digits at 1 year in study groups compared to 18 months in control group.

At the end of second year, 90% patients of study group achieved 2-point discrimination of <10 mm compared to only 60% in control group (*P* < 0.05).

One patient (5%) in the study group had 2-PD more than 15 compared to 5 patients (25%) in the control group (Table 2).

Semmes-Weinstein monofilament test showed a score of ≤2 in 8 patients (40%) in the study group compared to only 2 patients (10%) in the control group (*P* < 0.05) (Table 3).

Pain/discomfort for cold intolerance was higher in control group as compared to study group (Table 4).

Normal cold intolerance was noticed in 4 (20%) patients in study group as compared to 1 (5%) patients in control group.

In terms of motor recovery, 13 patients (65%) achieved satisfactory hand function (power 4/5) in study group as compared to 8 patients (40%) in control group (*P* < 0.05) (Table 5).

MRC scale was 2 or less than 2 in 85% of patients in study group compared to 60% in control group (Table 6).

The nerve conduction findings showed better results in study group as compared to control group (Table 7).

*Statistical Analysis.* All data analysis was conducted using SPSS software (SPSS Inc.) Significant differences were calculated using Fisher exact test, with *P* < 0.05 considered significant.

## 4. Discussion

Repair of large nerve gap remains a controversial issue. There are many options available for the reconstruction of these defects. Cable graft has long been considered an ideal option [4, 14–17]. However, continued research leads to the use of many alternative bridging materials in order to avoid donor nerve morbidity. These include the use of vein graft [18, 19], arterial grafts [20], and muscle graft for short nerve gap (less than 3 cm). These options are difficult to use for larger defects. Use of vein and muscle for larger defect may lead to collapse and dispersion of regenerating axons out of the muscle respectively.

Later on, filling of vein with muscle fibre and pieces of nerve was recommended to avoid the collapse of veins when used for large nerve gap [21]. Brunelli et al. report the application of vein conduit filled with muscle fibres in rat model for reconstruction of nerve gap. This technique leads to better functional outcome as compared to isolated vein conduit or muscle graft. Since its description, this method was not widely used for many years till Battiston et al. showed their clinical results on “Nerve repair by means of vein filled with muscle grafts” in 2004 and then they reviewed the literature later and described their clinical experience comparing biological and synthetic conduits for sensory nerve repair [22, 23].

Aly and Azab described a new technique using both cable nerve autograft and autogenous vein conduit to reconstruct wide nerve defects to get the benefits of both the methods [24].

In this series, we have used the similar technique of combined use of cable nerve graft and vein conduit to reconstruct the defects of more than 3 cm in size and compared the results with the use of isolated cable nerve graft technique. We noted a significantly better outcome in our study group where vein conduit was used as compared to control group where no conduit was used. This difference could be due to the prevention of sprouting of nerve fibre at the neurorraphy site by vein conduit in the study group.

Aly and Azab used the sural nerve and great saphaneous vein in their study. In our study we have used the sural nerve and short saphenous vein. This allowed us to harvest the nerve and vein through the same incisions leading to lower operating time and donor site scar as compared to Aly and Azab study.

Various researchers have demonstrated that for regeneration and maturation of nerve fibres, autogenous vein grafts are the supportive conduits [25, 26]. The advantages of veins grafts are that these are nonimmunogenic, easy to harvest, and available in variable sizes, have longer half-life, and are less inflammatory [27]. The vein wall is thin, acting as a barrier against scar tissue ingrowing and permeable enough to allow adequate nutrients diffusion and provide a favorable internal environment for nerve regeneration and maturation. Laminin, a glycoprotein, is found in good concentration in all the three layers of vein wall. Laminin promotes neurite and enhances nerve cell adhesion, proliferation, and differentiation thus helping to direct growth cone neurite [28].

There are various modalities for evaluation of nerve recovery. Wong et al. [29] assessed different tools for evaluation of peripheral nerve function. These include the touch moving 2-point discrimination (2PD); Semmes-Weinstein (SW) monofilament test, motor (Medical Research Council (MRC) scale), combined motor and sensory (Dellon modification of the Moberg pick up test; Moberg Recognition test), and pain (visual analogue scale; pinprick-test). They found that the results of the moving 2 PD were comparable with those of the SW monofilaments but having poor correlation and MRC score correlated well with opposition movement of the thumb and muscle wasting (*P* < 0.01). We used 2 PD, Semmes-Weinstein (SW) monofilament test for sensory evaluation and power of the involved muscle and MRC for evaluation of motor recovery.

Aly and Azab found that majority of the patients in their series had adequate and useful 2-PD (60% with 2-PD < 10 mm) and over 82% resumed light touch with variable degrees. The results were comparable to our study where 90% of the patients resumed 2 PD < 10 mm and 85% had SW monofilament score ≥3. In terms of motor recovery, 85% of patients in our study group resumed a power of ≥3 leading to good functional gain.

## 5. Conclusion

It was concluded that the use of vein conduit in peripheral nerve repair is more effective method than isolated nerve graft providing good sensory and motor recovery.

## Figures and Tables

**Table 1:** Baseline characteristics.

Characteristics	Study group = 20 (nerve graft + vein conduit)	Control group = 20 (nerve graft)
Mean age (years)	28 ± 7	29 ± 6
Etiology	Sharp injury—8 (40%) Crush injury—12 (60%)	Sharp injury—9 (45%) Crush injury—11 (55%)
Types of nerve	Ulnar—7 Median—13	Ulnar—8 Median—12
Time since injury	Old injury—5 (25%) Fresh injury—15 (75%)	Old injury—6 (30%) Fresh injury—14 (70%)
Nerve defect	3.5 cm—6 (30%) 4.5 cm—8 (40%) 5.5 cm—6 (30%)	3.5 cm—7 (35%) 4.5 cm—7 (35%) 5.5 cm—6 (30%)
Types of repair	Epineural	Epineural
Mean distance from proximal nerve end to tip of middle finger	10–15—5 15–20—13 >20—2	10–15 cm—6 15–20 cm—12 >20 cm—2
Smoking	3 (15%)	4 (20%)
Associated vascular injury	4 (20%)	3 (15%)
Associated tendon injury	3 (15%)	4 (20%)
Associated co morbidity	None	None

**Table 2:** Sensory outcome—2 PD.

2 PD (mm)	Study group Number of patients (%)	Control group Number of patients (%)
<5	7 (35%)	4 (20%)
5–10	11 (55%)	8 (40%)
10–15	1 (5%)	3 (15%)
>15	1 (5%)	5 (25%)

*P* < 0.05.

**Table 3:** Sensory outcome—SW exam.

Semmes Weinstein monofilament test	Study group Number of patients (%)	Control group Number of patients (%)
1	3 (15%)	1 (5%)
2	5 (25%)	1 (5%)
3	9 (45%)	8 (40%)
4	3 (15%)	10 (50%)

*P* < 0.05.

**Table 4:** Pain/discomfort evaluation—cold intolerance.

Scale (problem estimation)	Study group Number of patients	Control group Number of patients
0	2 (10%)	6 (30%)
1	3 (15%)	7 (35%)
2	11 (55%)	6 (30%)
3	4 (20%)	1 (5%)

**Table 5:** Motor outcome—power.

Power grade	Study group Number of patients	Control group Number of patients
0	0 (0%)	3 (15%)
1	0 (0%)	3 (15%)
2	3 (15%)	3 (15%)
3	4 (20%)	3 (15%)
4	10 (50%)	6 (30%)
5	3 (15%)	2 (10%)

*P* < 0.05.

**Table 6:** Motor outcome—MRC scale.

MRC scale	Study group Number of patients	Control group Number of patients
0	8 (40%)	2 (10%)
1	5 (25%)	3 (15%)
2	4 (20%)	7 (35%)
3	3 (15%)	8 (40%)

**Table 7:** Nerve conduction findings.

Characteristics	Study group	Control group
Conduction velocity	Normal—14 (70%) Decreased—6 (30%)	Normal—7 (35%) Decreased—13 (65%)
Latency	Normal—19 (95%) Increased—1 (5%)	Normal—10 (50%) Increased—10 (50%)
Amplitude	Normal—14 (70%) Increased—6 (30%)	Normal—8 (40%) Increased—12 (60%)
